# Endovascular repair with the Gore thoracoabdominal multibranch endoprosthesis for proximal degeneration after prior fenestrated endovascular aortic repair

**DOI:** 10.1016/j.jvscit.2024.101664

**Published:** 2024-10-26

**Authors:** Lauren Cralle, Kathryn DiLosa, Steven Maximus

**Affiliations:** Division of Vascular Surgery, University of California Davis Medical Center, Sacramento, CA

**Keywords:** Thoracoabdominal aneurysm, Endovascular repair, Proximal degeneration

## Abstract

Degeneration of the thoracoabdominal aorta proximal to a prior fenestrated endovascular aortic repair represents a complex issue with limited options for repair. Previously, modified endografts or open conversion with endograft explant offered the only options for management. Here we describe use of the Gore Thoracoabdominal Multibranch Endoprosthesis for exclusion of an extent III thoracoabdominal aneurysm in the setting of degeneration proximal to a previously placed fenestrated device.

Since the US Food and Drug Administration (FDA) approval of endovascular aortic aneurysm repair (EVAR) in 1999, the management of abdominal aortic aneurysms has evolved from primarily open operative interventions to increasingly complex endovascular techniques, including adjuncts like the iliac branch endoprosthesis and the conformable Excluder (W. L. Gore & Associates, Newark, DE). Similarly, increasingly complex juxtarenal and thoracoabdominal aortic aneurysms are becoming amenable to repair. Initially such repairs were accomplished via off-label modifications of existing devices (physician-modified endografts) and parallel grafting techniques such as snorkel or chimney configurations, then later with on-label custom grafts with fenestrations specifically designed to incorporate visceral branches (Zenith Fenestrated Endovascular Aortic Repair [ZFEN]; Cook Medical, Bloomington, IN).^1^ With all of these tools, more complex pathologies were able to be repaired surgically via an endovascular approach and higher risk patients could be managed more safely electively, with decreased morbidity and mortality of endovascular repair as compared with traditional open surgical options.^2^

With the more widespread adoption of these endovascular repairs, more patients are at risk of proximal aneurysmal degeneration.^3^^,^^4^ Both FDA approved and off-label modifications require some degree of healthy aortic tissue for a proximal seal zone, which allows for adequate anchoring and positioning of the endograft and mitigates further aneurysmal degeneration by excluding the diseased aorta.^5^^,^^6^ One option for the management of proximal disease progression includes conversion to open repair, which has high associated morbidity and mortality owing to explantation of the prior endograft and debranching of visceral vessels with a supraceliac or suprarenal aortic cross clamp.^7^ Alternatively, prior endovascular options have included off-label techniques, such as custom physician-modified endograft, which carries its own risk of morbidity and mortality and can prove challenging technically.^8^

As of January 2024, the Gore Excluder thoracoabdominal multibranch endoprosthesis (TAMBE) (W. L. Gore & Associates) gained FDA approval, offering the first off-the-shelf device for repair in thoracoabdominal aneurysms. The TAMBE has four built-in, precannulated internal portals that allow bridging of visceral stents to the abdominal vessels in a noncustomized fashion, amenable to various anatomic configurations.^9^

This case report, with patient permission, describes an alternative endovascular technique for the repair of paravisceral aortic degeneration in the setting of prior fenestrated aortic endograft, using the TAMBE device.

## Case report

**Fig 1:**
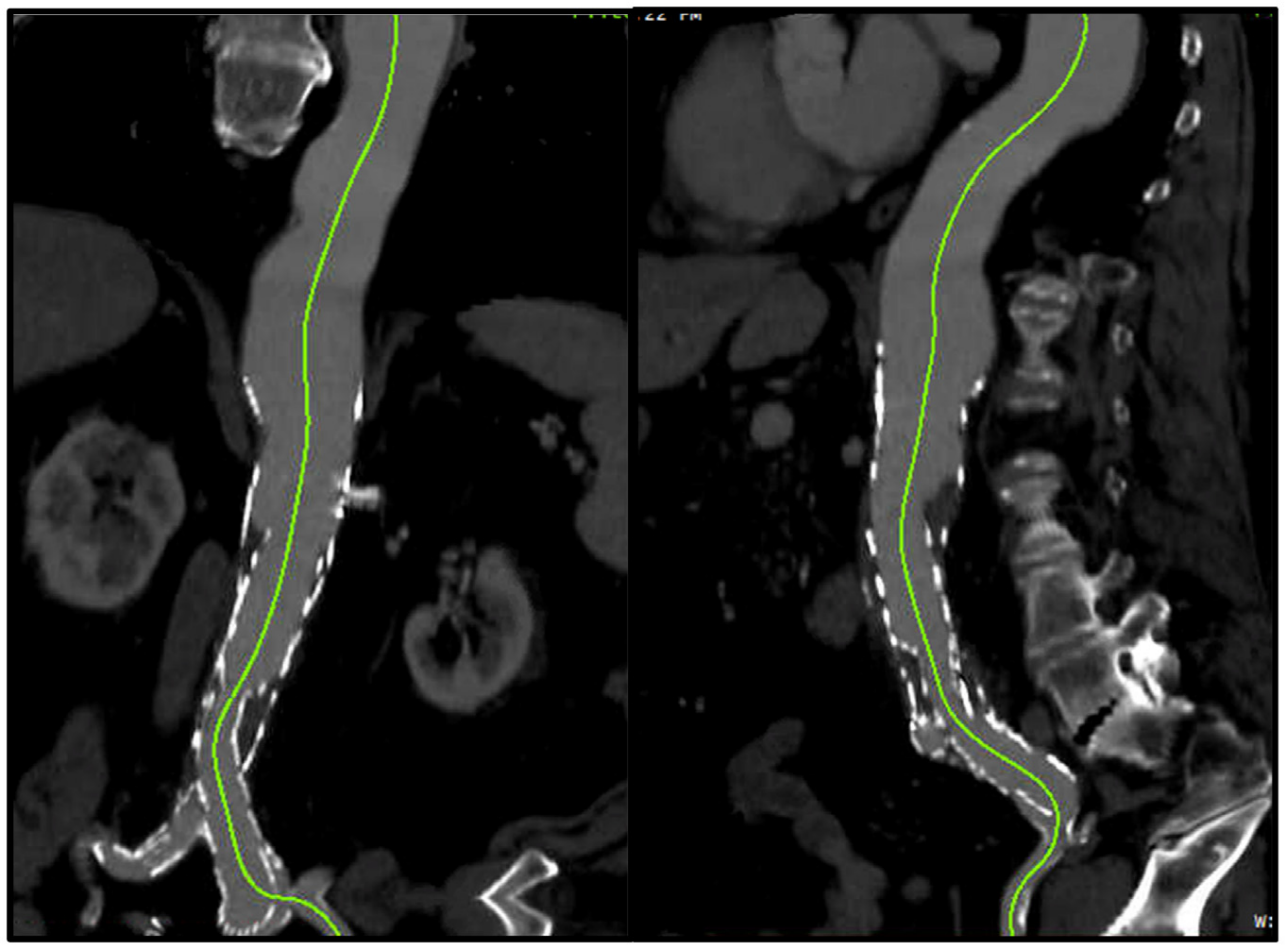
Preoperative centerline imaging of prior fenestrated repair.

The patient was a 76-year-old man with a past medical history of hypertension, chronic obstructive pulmonary disease, atrial fibrillation on apixaban (Eliquis), former tobacco use, chronic kidney disease, and chronic left leg weakness and numbness owing to prior femoral nerve injury. He had undergone fenestrated endograft with bilateral renal artery stents and superior mesenteric artery scallop in 2016 at an outside facility. During outpatient surveillance, he was noted to have degeneration of the aorta proximal to the level of his fenestrated repair, now with an extent III thoracoabdominal aneurysm, with a maximal diameter of 5.8 cm at the level of the celiac artery (Fig 1). With the prior fenestrated repair and baseline kidney disease, open conversion carried a high risk of perioperative morbidity and worsening kidney function. The patient was counseled extensively on the risks and benefits of open and endovascular repair options, including spinal cord ischemia, renal failure, mesenteric ischemia, and limb ischemia given the complex nature of the proposed complex endovascular repair. With shared decision-making between the surgeon and patient, repair with the TAMBE device was deemed the safest and most effective option.

The prior fenestrated device measured 36 mm in diameter and 137 mm in length, and a 91-mm bifurcated distal main body component was sealed into the left common iliac artery, with a 13 mm Zenith Spiral iliac limb (Cook Medical) into the right common iliac artery with the standard three-stent overlap. Both renal arteries were cannulated with 6 mm iCAST stents (Atrium Medical Corporation, Merrimack, NH). The native left renal artery was noted to have stenosis just distal to the prior stent; thus, to maximize the seal to prevent a late type IC endoleak and to treat the stenosis, a plan for seal distal to this stenotic area was planned. To fully exclude the new aneurysm, a seal zone approximately 8.5 cm above the celiac origin was identified, measuring 34 mm in diameter, and a 37-mm Gore TAMBE device was chosen for repair.

After successful induction of anesthesia, the patient was positioned with arms above the head to minimize radiation. The surgical team then proceeded with obtaining percutaneous bilateral common femoral artery and percutaneous right axillary artery access. Therapeutic heparin was administered, and through-and-through wire access was obtained with a 300-cm Metro wire (Cook Medical). The TAMBE device was positioned appropriately having used the Gore tri-lumen catheter to precannulate the portals. The device was delivered through a 22F dry seal sheath introduced through the right common femoral artery and positioned at the level of the paravisceral aorta. An 8F sheath was introduced in the left common femoral artery. In the standard fashion, the native celiac and super mesenteric arteries were cannulated without difficulty and the bilateral renal arteries were easily cannulated through the prior iCAST stents. The device was deployed and all visceral vessels were stented accordingly. A 7-mm Viabahn Balloon-Expandable (VBX) stent (W. L. Gore & Associates) was sealed into the celiac artery with a 9-mm VBX bridging stent. Similarly, a 7-mm VBX stent was sealed into the superior mesenteric artery with additional 7-mm bridging stents. Within the bilateral renal arteries, 5-mm VBX stents were sealed into the target vessels with 7-mm VBX bridging stents.

**Fig 2:**
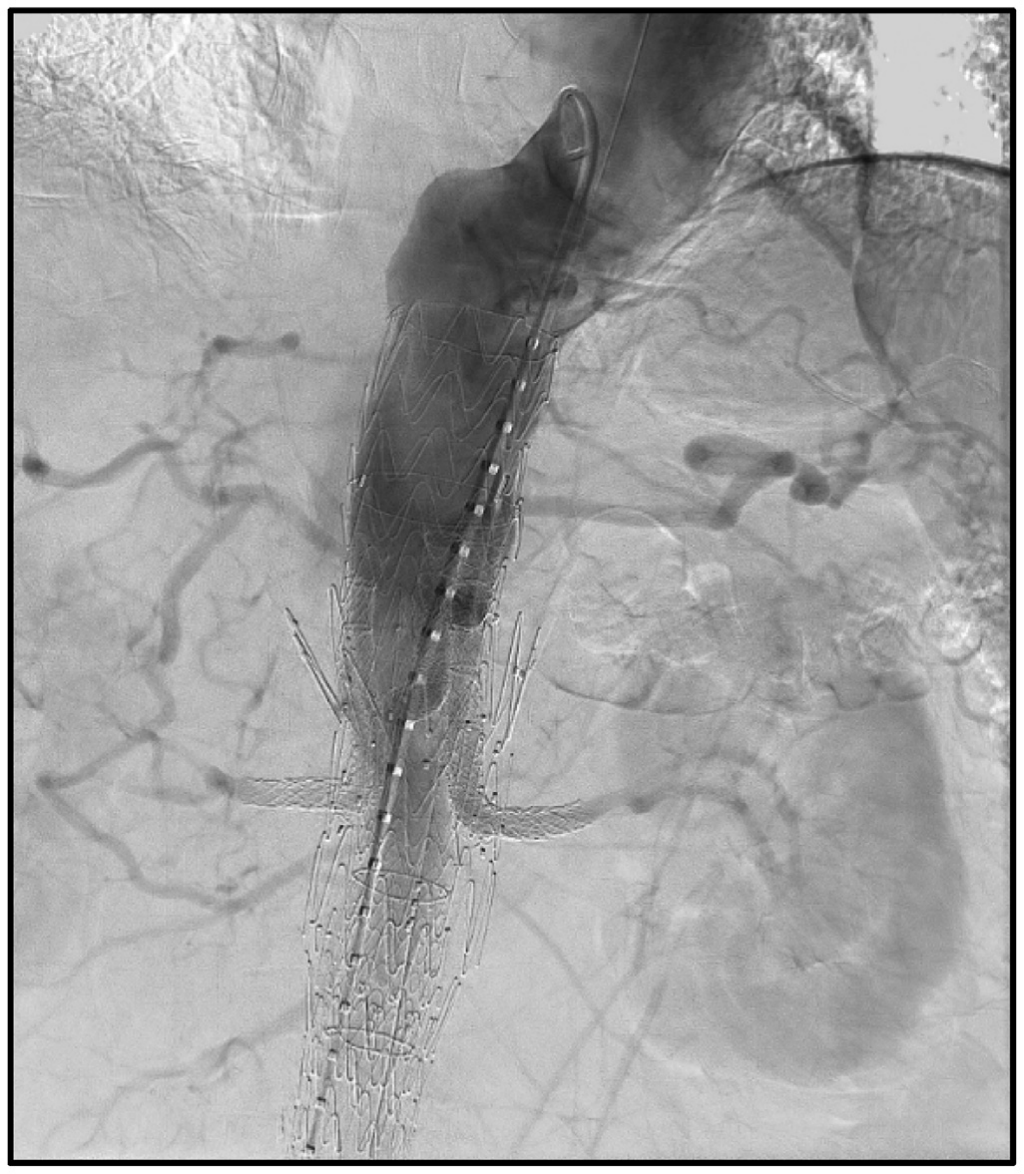
Completion imaging after cannulation and deployment of proximal graft into prior fenestrated endovascular aortic aneurysm repair (EVAR).

Completion imaging after this portion of the procedure demonstrated patent visceral stents; however, there seemed to be a type IA endoleak (Fig 2). Intravascular ultrasound examination revealed the thoracic aorta to be 35 to 38 mm proximal to the TAMBE device, so the decision was made to place a 45 mm × 10 cm Conformable Thoracic Aortic Graft (W. L. Gore & Associates) to obtain adequate seal into zone 4 of the descending thoracic aorta with complete resolution of the endoleak. The remainder of the procedure was completed without incident and a 23 mm × 14.5 mm × 10 cm iliac branch device was sealed into the TAMBE device. Distally, an 8L VBX was deployed on the left along with two 14-mm Excluder iliac limbs (W. L. Gore & Associates) and an additional 8L VBX on the right.

**Fig 3:**
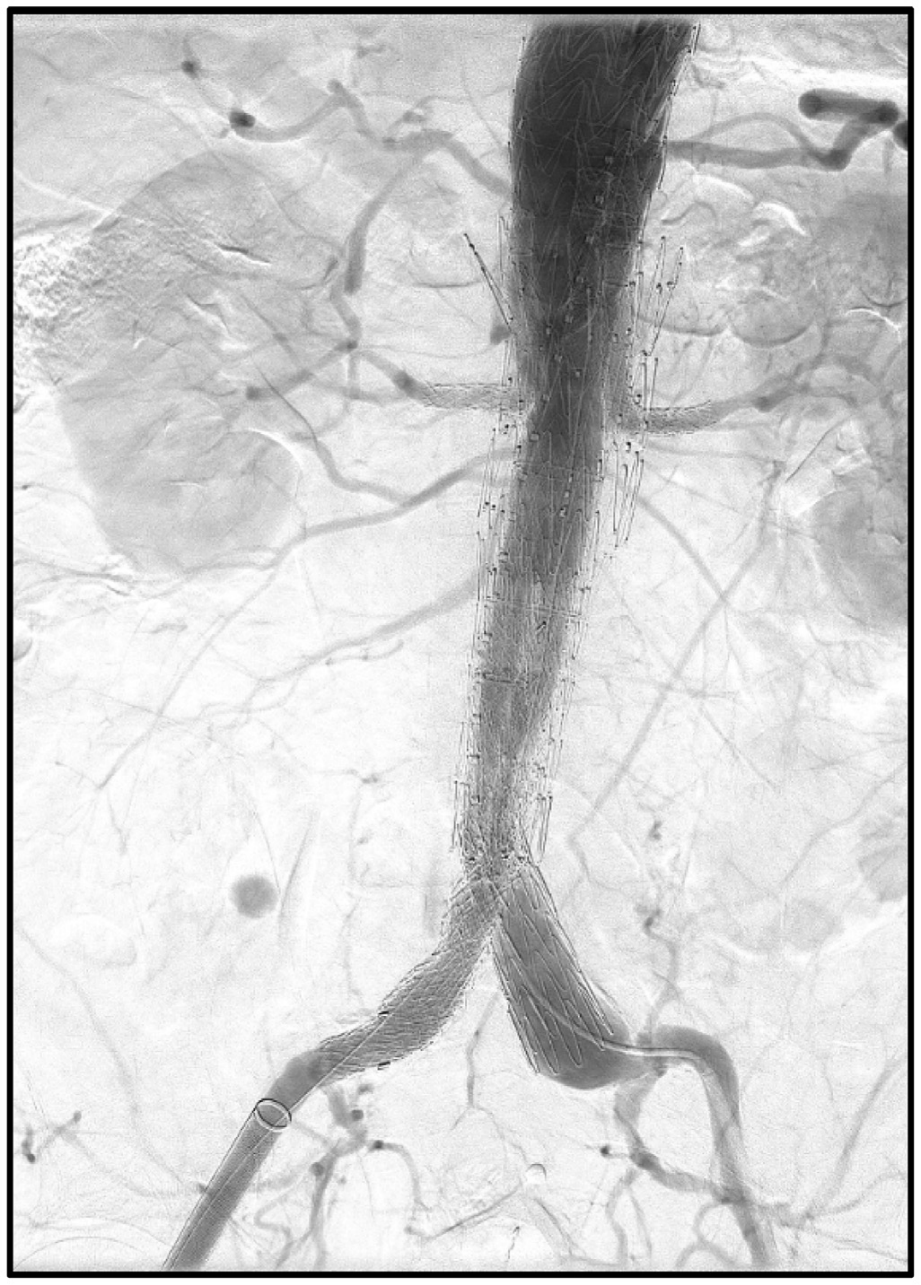
Final case completion imaging, showing patent bilateral renal artery stents and no evidence of a distal endoleak.

Overlapping stent segments were post-dilated with the Molding and Occlusion Balloon (W.L. Gore & Associates) in the infrarenal portions of the device. A final completion angiogram revealed no evidence of endoleak and filling of the celiac, superior mesenteric, and bilateral renal artery branches (Fig 3). Access sites were closed with preplaced closure devices and the patient was extubated successfully and taken to the recovery unit.

The patient had an uneventful postoperative course. He was monitored in the intensive care unit for 48 hours and was discharged home postoperative day 3. Surveillance imaging completed at 6 weeks demonstrated patent visceral stents and exclusion of the aneurysm sac without evidence of endoleak.

## Discussion

Endovascular aortic repair options have become increasingly complex with the introduction of new devices. The advent of the ZFEN device facilitated aortic repair with a 4-mm infrarenal neck, offering a durable repair for patients without adequate infrarenal seal. Although the ZFEN device may exclude an infrarenal aneurysm successfully, it does not prevent aneurysmal degeneration of the paravisceral aorta proximal to this device. Degeneration of this aortic segment may eventually reach a threshold where repair is indicated or may compromise the seal of the fenestrated device resulting, in a type IA endoleak.

Previously described options for repair in such settings are limited. Open conversion with explant of a fenestrated aortic device is associated with an increased risk of morbidity and mortality, especially with the explantation of visceral stents. Endovascular repair options remain complex, frequently requiring parallel grafting or custom endograft modification. Custom medical devices, including the ZFEN+ (Cook Medical) could be designed specifically to address the complex anatomy, although access to these devices is currently limited to those surgeons with an FDA investigational device exemption. The TAMBE device offers an off-the-shelf alternative for endovascular repair in these complex patients.

Surgeon familiarity with the ZFEN device for the repair of juxtarenal aneurysms has led to ready adoption of this approach when a short neck makes traditional EVAR unlikely to offer a durable aneurysm repair. A consequence of the more widespread adoption of fenestrated repairs is increased complexity when proximal degeneration occurs. Although considered off label, the TAMBE device can be sealed successfully into the supraceliac aorta with relining of the infrarenal component.

There are some important considerations when evaluating a patient for the use of the TAMBE device in the setting of a prior fenestrated repair. The TAMBE device requires a diameter of ≥20 mm within the visceral segment for device deployment and sufficient working room for cannulating the target vessels. Consideration of the diameter of prior devices is necessary to ensure adequate expansion of the device and associated branches. Most important, an adequate landing zone proximal to the bifurcated distal main body ZFEN device is imperative to avoid inadvertent conversion into an aortouni-iliac repair that would then require revascularization of the devascularized extremity. Han et al^10^ have described this as an important consideration in failed EVAR previously, observing that the TAMBE device may be required to seal more proximally than would otherwise be required to facilitate landing the device at least a centimeter above the prior device bifurcation. We similarly observed that the seal required more extensive aortic coverage than would have been otherwise required, although it was necessary to allow for cannulation for contralateral iliac limb.

Additionally, the tapered portion of the TAMBE device is unlikely to obtain an adequate seal in a prior device, necessitating that the infrarenal aorta and iliac vessels be relined to prevent subsequent endoleak. Although in the presence of multiple prior devices, visualization for relining of the pararenal and infrarenal aortic segments may be compromised. Close collaboration with industry partners familiar with both the TAMBE and ZFEN devices is recommended.

## Conclusions

In the setting of proximal aortic aneurysmal degeneration following prior fenestrated EVAR, this report confirms that use of the off-the-shelf Gore TAMBE device offers a viable endovascular repair option.

## Disclosures

None.
